# Understanding the Demographic, Clinical, and Real-Time Polymerase Chain Reaction Profiles of COVID-19 Patients in a Tertiary Care Hospital in Northeast India

**DOI:** 10.7759/cureus.35426

**Published:** 2023-02-24

**Authors:** Bornali S Dutta, Kalyan Nath, Manash J Taw, Ajanta Sharma, Gitika Rajbongshi, Kailash Chamuah, Nungshi Henbi, Rinku K Barman, Santhalembi Chingtham, Derhasar Brahma, Kimmi Sarmah, Purabi Baruah, Kripamay Nath, Parasmita D Choudhury, Dikshita Mazumder, Abhijit Sarmah, Anupal Sharma, Basanta Hazarika, Manoj K Choudhury, Achyut C Baishya

**Affiliations:** 1 Microbiology, Gauhati Medical College and Hospital, Guwahati, IND; 2 Medicine, Gauhati Medical College and Hospital, Guwahati, IND; 3 Surgery, Gauhati Medical College and Hospital, Guwahati, IND; 4 Physiology, Gauhati Medical College and Hospital, Guwahati, IND; 5 Pulmonary Medicine, Gauhati Medical College and Hospital, Guwahati, IND; 6 Community Medicine, Gauhati Medical College and Hospital, Guwahati, IND

**Keywords:** sars-cov-2, demographic profile, covid-19, rt-pcr, clinical profile

## Abstract

Introduction and aims

The demographic and clinical profile and dynamics of real-time polymerase chain reaction (RT-PCR) in coronavirus disease 2019 (COVID-19) patients are not well understood. The study aimed to analyze the demographic, clinical, and RT-PCR profiles of COVID-19 patients.

Methodology

The study was a retrospective, observational study conducted at a COVID-19 care facility, and the study period was from April 2020 to March 2021. Patients with laboratory-confirmed COVID-19 by real-time polymerase chain reaction (RT-PCR) were enrolled in the study. Patients with incomplete details or with only single PCR tests were excluded. Demographic and clinical details and the results of severe acute respiratory syndrome coronavirus 2 (SARS-CoV-2) RT-PCR collected at different time points were retrieved from the records. The statistical software Minitab version 17.1.0 package (Minitab, LLC, State College, PA, USA) and Rstudio version 1.3.959 (Rstudio, Boston, MA, USA) were used for the statistical analysis.

Results

The mean duration from symptom onset to the last positive RT-PCR was 14.2 ± 4.2 days. The proportions of positive RT-PCR tests were 100%, 40.6%, 7.5%, and 0% at the end of the first, second, third, and fourth weeks of illness. The median duration of days to first negative RT-PCR in the asymptomatic patients was 8 ± 4 days, and 88.2% of asymptomatic patients were RT-PCR-negative within 14 days. A total of 16 symptomatic patients had prolonged positive test results even after three weeks of symptom onset. Older patients were associated with prolonged RT-PCR positivity.

Conclusion

This study revealed that the average period of RT-PCR positivity from the onset of symptoms is >2 weeks in symptomatic COVID-19 patients. Prolonged observation in the elderly population and repeat RT-PCR before discharge or discontinuation of quarantine is required.

## Introduction

During the last two decades, several viral epidemics due to coronaviruses have occurred, such as the severe acute respiratory syndrome coronavirus (SARS-CoV) from 2002 to 2003 and, most recently, the Middle East respiratory syndrome coronavirus (MERS-CoV), which was first identified in Saudi Arabia in 2012. Within a few months of reporting of severe acute respiratory syndrome coronavirus 2 (SARS-CoV-2) infection in Wuhan City, China, in December 2019, it engulfed almost all countries in the world, where the USA, Europe, and Southeast Asia bore the maximum brunt of disease load [1]. A total of 281,808,270 confirmed cases have been detected globally, with over 5,411,759 deaths occurring at the end of December 29, 2021, as declared by the World Health Organization (WHO) [2]. In India, the first case of coronavirus disease 2019 (COVID-19) was identified on January 30, 2020, and the number has been increasing steadily due to local transmission and foci of community transmission [3]. As of December 29, 2021, the number of cases in India was 34,808,886, with an overall reported mortality of 480,592 [2]. The first case of COVID-19 in Assam was reported on March 31, 2020, from the Karimganj district. The COVID-19 outbreak in Assam has been traced to persons who attended a religious congregation in Delhi. Assam recorded 612,282 cases until December 31, 2021, with 6,164 deaths [4].

The ongoing pandemic poses many clinical and public health management challenges due to a limited understanding of viral pathogenesis, risk factors for infection, the natural history of disease including clinical presentation and outcomes, prognostic factors for severe illness, period of infectivity, modes and extent of virus interhuman transmission, and effective preventive measures and public health response and containment interventions. It is evident from various studies that the demographic profile, clinical presentation, and outcome of patients with COVID-19 vary in different countries and also within India [5-8]. SARS-CoV-2 detection at different points of time during the course of infection, including in asymptomatic patients, will help interpret the real-time PCR (RT-PCR) test results with regard to the period of viral shedding. Understanding the dynamic profile of SARS-CoV-2 in patients’ respiratory specimens can help in diagnosing COVID-19 and reflecting the disease course, which in turn will help in the management of the pandemic. The epidemiological, demographic, and clinical features of this contagious virus have started to appear in the literature. Various studies have reported the detection of SARS-CoV-2 by RT-PCR at different time points, especially during the acute phase of infection. Moreover, no study has been reported specifically from Northeast India regarding the effect of the demographic and clinical factors on the RT-PCR profile of SARS-CoV-2 during a long observation period. Therefore, this study aimed to analyze the demographic, clinical, and dynamic profiles of SARS-CoV-2 infection and its outcome and explore the impact of demographic and clinical parameters on it.

## Materials and methods

Study design

The study was a retrospective, observational study conducted at the COVID-19 care facility under Gauhati Medical College and Hospital, Guwahati, Assam, India.

Study period

The data of the study participants admitted from April 2020 to March 2021 were retrieved from the hospital electronic database and included in the study.

Ethical approval

The study was approved by the Institutional Ethics Committee vide letter number MC/190/2007/Pt-II/DEC-2020/13 (dated 12.01.2021).

Study participants

Five hundred consecutive patients with or without symptoms with RT-PCR result positive for COVID-19 and admitted to a dedicated COVID-19 facility (symptomatic patients of all severity) and quarantine facility (asymptomatic patients who were in direct contact with positive cases) of the tertiary care hospital were included.

Collection of patient samples

Both nasal and oropharyngeal swabs were collected using viral transport media (VTM) from HiMedia (Mumbai, India) on the arrival of the patients in the screening area of the hospital.

RT-PCR assay

Viral RNA was extracted using QIAamp Viral RNA Mini Kit (Qiagen, Hilden, Germany). The RT-PCR assay for SARS-CoV-2 was performed by using the Indian Council of Medical Research (ICMR) recommended kits. The diagnostic criterion was based on the recommendation of the National Institute of Virology, Pune. Cases with laboratory-confirmed diagnosis of COVID-19, made by positive SARS-CoV-2 RT-PCR on nasopharyngeal samples, were enrolled regardless of symptomatology. Patients with at least two SARS-CoV-2 RT-PCR reports were included in the study.

Data collection

Clinical information and results of RT-PCR for SARS-CoV-2 viral nucleic acid detection of the patients admitted from April 2020 to March 2021 were obtained from the electronic medical records system. The information recorded included demographic data, underlying comorbidities, clinical features, date of first and subsequent PCR tests and their results, treatment measures, and outcomes. We collected data on testing dates and RT-PCR results up until discharge. Additionally, we collected data on clinical outcomes, discharge dates, deaths, and length of hospital stay.

Classification of disease

The severity of the disease has been classified as per the Revised Guidelines on Clinical Management of COVID-19 of the Ministry of Health and Family Welfare, Directorate General of Health Services, Government of India (May 24, 2021) [9]. Patients with uncomplicated upper respiratory tract infection who may have mild symptoms such as fever, cough, sore throat, nasal congestion, malaise, and headache were considered as mild (without shortness of breath or hypoxia (normal saturation)). Patients with pneumonia with no signs of severe disease were considered as moderate (adults with clinical features of dyspnea and/or hypoxia, fever, cough, oxygen saturation (SpO2) of 90% to ≤93% on room air, and respiratory rate of more or equal to 24 breaths/minute). Patients with severe pneumonia were considered as severe cases (adults with clinical signs of pneumonia plus one of the following: respiratory rate > 30 breaths/minute, severe respiratory distress, and SpO2 < 90% on room air) [9].

Statistical analysis

The statistical software Minitab version 17.1.0 package (Minitab, LLC, State College, PA, USA) and Rstudio version 1.3.959 (Rstudio, Boston, MA, USA) were used for the statistical analysis. Descriptive statistics were used for reporting the mean, standard deviation (SD), variance, etc. under various categories and subcategories. The mean ± standard deviation was given for continuous variables. Continuous variables with extreme value (skewed) were presented as median and interquartile range (IQR), and categorical variables were reported as whole numbers. Box plot analysis was done to show the distribution of data on the basis of five number summary (“minimum,” first quartile (Q1), median, third quartile (Q3), and “maximum”). Data normality was checked using a normal probability plot. Empirical cumulative distribution function (CDF) plot analysis was done for continuous variables to obtain the percentile and proportion of data ranges.

One-way analysis of variance (ANOVA) was used to compare the differences in means between groups in a univariate analysis. Two-way ANOVA and three-way ANOVA were used in multivariable analysis to model the relationship between RT-PCR positivity days, age group, sex, and comorbidities of both symptomatic and asymptomatic patients. Population variation was considered significant at p value of <0.05. Interaction plot analysis was done to analyze the interaction between different groups. ANOVA test was followed by the homogeneity variance test (Levene’s test) to check for equality of variances. To meet the assumption of homogeneity of variance, the p-value for Levene’s test was considered below 0.05.

## Results

Demographic details

During the study period, five hundred patients were enrolled for the study. A total of 186 (37.2%) patients were symptomatic, and 314 (62.8%) were asymptomatic. Males predominated the study population, with 370 (74%), and there were 130 (26%) females, with a male-to-female ratio of 2.8:1. Most of the cases (56.5%) belonged to the age group of 21-40 years, followed by the age group of 40-60 years (20.2%). Other demographic details are shown in Table 1. The mean age of the symptomatic cases was 41.9 years (range: 9-86 years), and that of the asymptomatic cases was 35.4 years (range: 9-100 years).

**Table 1:** Descriptive statistics of the symptomatic and asymptomatic patients

Variables	Total patients	Symptomatic		Asymptomatic	
		Number	%	Number	%
	500	186	37.2	314	62.8
Gender					
Male	370	103	27.8	267	72.1
Female	130	83	63.8	47	36.1
Age group					
<20 years	81	16	19.7	65	80.2
21-40 years	284	79	27.8	205	72.1
41-60 years	102	64	62.7	38	37.2
>60 years	33	27	81.8	6	18.1
Comorbidities					
Hypertensive	78	46	24.7	32	10.2
Non-hypertensive	422	140	75.2	282	89.8
Diabetic	48	32	17.2	16	5
Non-diabetic	452	154	82.8	298	94.9
Malignancy	7	4	2.1	3	0.95
Without malignancy	493	182	97.8	311	99

Clinical details

Of the symptomatic patients, 152 (81.7%) were mild cases, 17 (9.13%) were moderate, and 17 (9.13%) were severe. Among the predominant symptoms, fever was the commonest presenting symptoms (57.5%), followed by cough (43%), breathing difficulty (22.5%), generalized weakness (11.8%), headache (7.5%), diarrhea (4.8%), sore throat (3.7%), anosmia (3.7%), and chest pain (3.2%). Overall, 46 (85.2%) patients with severe disease and 30 (24.8%) with moderate disease required admission into the intensive care unit (ICU). The SpO2 of severe cases ranged from 32% to 90%, and that of moderate cases from 82% to 99%.

Eighty-five (45.7%) of the symptomatic cases and 263 (83.8%) of the asymptomatic cases did not have any comorbidities. In both symptomatic and asymptomatic cases, hypertension was the most common comorbidity (24.7% and 10.2%, respectively), followed by diabetes mellitus (17.2% and 5%, respectively). Malignancy was seen in 2.1% of the symptomatic and 0.95% of the asymptomatic cases (Table 1), and 19 (18.8%) had other comorbidities such as hypothyroidism, chronic kidney disease, and chronic obstructive pulmonary disease (COPD). Among the severe cases, six (35.2%) had multiple comorbidities such as type 2 diabetes mellitus and hypertension, four (23.5%) had only hypertension, three (17.6%) had only type 2 diabetes mellitus, and one (5.2%) had hypertension, type 2 diabetes mellitus, and hypothyroidism. Among the moderate cases, the most common comorbidity was hypertension (58.8%), followed by type 2 diabetes mellitus (41.1%).

RT-PCR profile of SARS-CoV-2 infection

For 500 patients, a total of 2,088 RT-PCR tests were done, with an average of 4.16 tests per patient. Of the 500 patients, 451 (90.2%) patients (137 symptomatic and 314 asymptomatic) recovered and were discharged at the time of the final analysis. Forty-nine (9.8%) patients died during the hospital stay.

**Figure 1:**
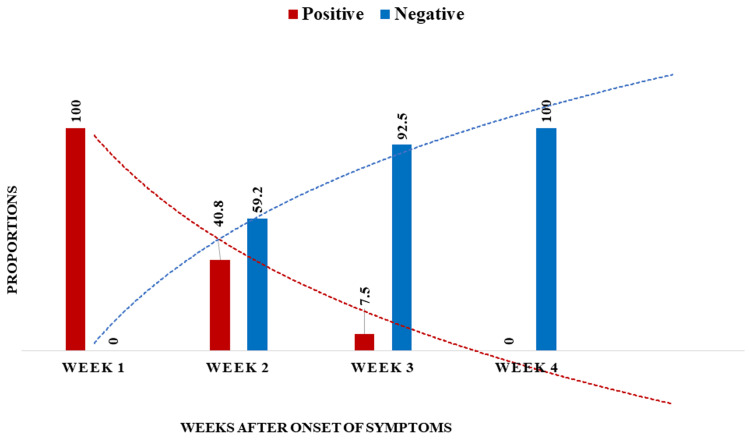
Dynamic profile of SARS-CoV-2 RT-PCR in symptomatic cases SARS-CoV-2: severe acute respiratory syndrome coronavirus 2, RT-PCR: real-time polymerase chain reaction

The mean duration from onset of symptoms to the first positive RT-PCR was 4.7 ± 3.0 days, and the median duration was four (IQR: 2-15) days. The mean duration from onset of symptoms to the last positive RT-PCR was 14.2 ± 4.2 days, and the median duration was 13 (IQR: 9-25) days. In the asymptomatic patients, the mean duration from the first positive RT-PCR to the last positive RT-PCR was 8 ± 4 days, and the median duration was eight (IQR: 4-20) days. The median duration of time between the first and the last positive RT-PCR tests in symptomatic and asymptomatic patients were 11 (IQR: 9-25) and 9 (IQR: 4-20) days, respectively. The details of the dynamic profile of the SARS-CoV-2 RT-PCR of 186 symptomatic patients is shown in Figure 1.

At the end of the first week after the onset of symptoms, all patients showed positive results for RT-PCR for SARS-CoV-2. Second week onward, the RT-PCR test negativity increased. At week 4 after onset, all RT-PCR test results were negative (Figure 1). The RT-PCR positivity was highest (100%) at week 1, followed by weeks 2, 3, and 4 (40.8%, 7.5%, and 0%, respectively).

Impact of demographic and clinical factors on the RT-PCR profile of SARS-CoV-2 infection

**Figure 2:**
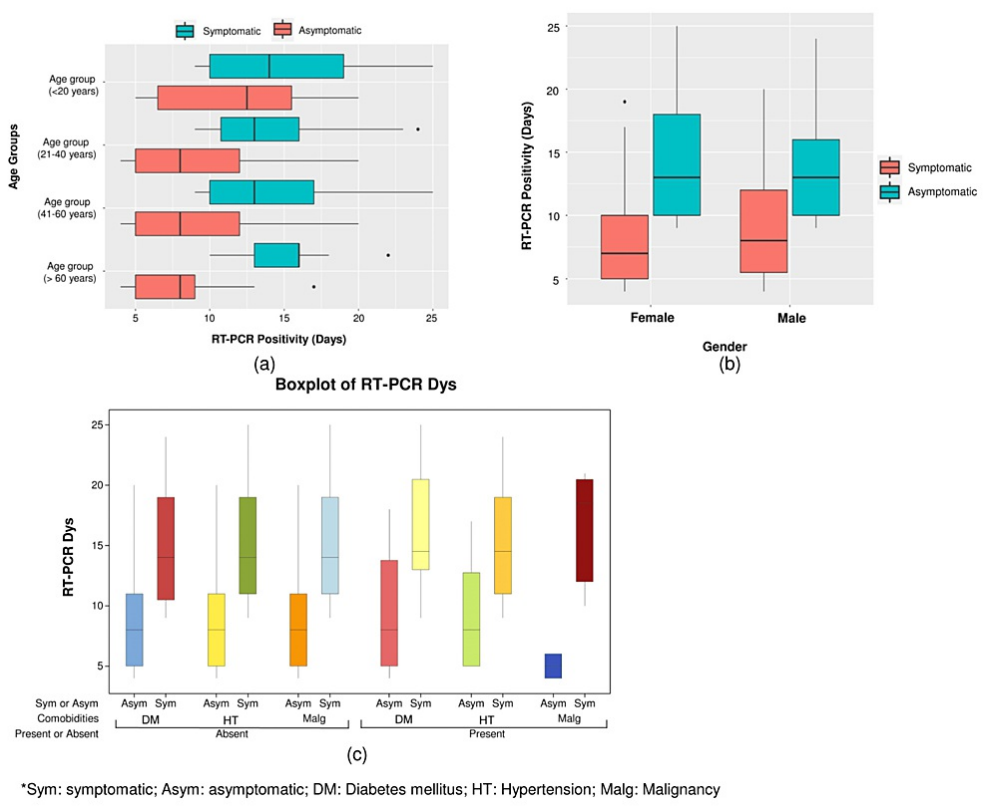
Box plot analysis of RT-PCR positivity of (a) different age groups, (b) different genders, and (c) different comorbidities in symptomatic and asymptomatic patients RT-PCR: real-time polymerase chain reaction, Sym: symptomatic, Asym: asymptomatic, DM: diabetes mellitus, HT: hypertension, Malg: malignancy

Among the symptomatic patients, the mean duration of RT-PCR positivity in the age group of >60, 41-60, 21-40, and ≤20 years were 14.8 ± 4.5, 14.1 ± 4.1, 14.0 ± 4.3, and 14.9 ± 3.8 days, respectively. Among the asymptomatic patients, the mean durations of positivity from the first RT-PCR to the last RT-PCR were 7.8 ± 2.9, 9.3 ± 4.1, 9.3 ± 4.8, and 11.8 ± 6.0 among the age groups of ≤20, 20-40, 40-60, and >60 years, respectively. The duration of RT-PCR positivity of >3 weeks was highest in the age group >60 years. The box plot analysis of the duration of RT-PCR positivity of different age groups in symptomatic and asymptomatic patients is presented in Figure 2a.

**Figure 3:**
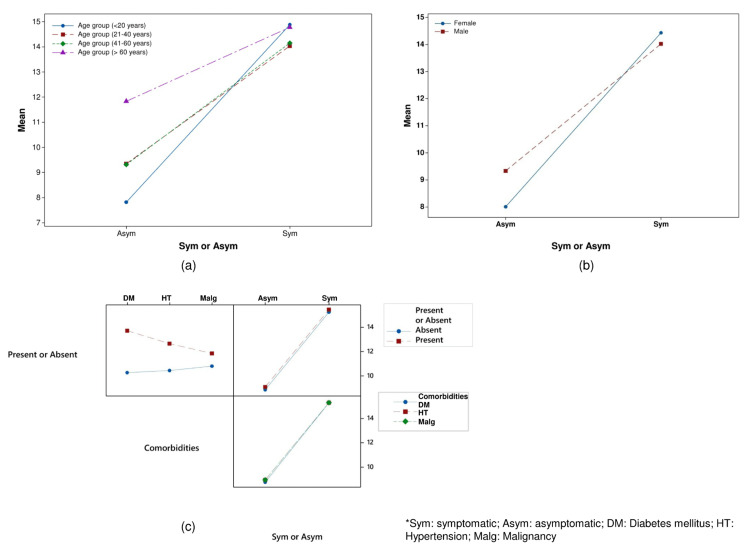
Interaction plot analysis of (a) age groups, (b) gender, and (c) comorbidities Sym: symptomatic, Asym: asymptomatic, DM: diabetes mellitus, HT: hypertension, Malg: malignancy

One-way ANOVA test showed that in symptomatic patients, there was no significant variation among the different age groups (F = 0.3407, p = 0.79), but in asymptomatic patients, significant variation was observed among the age groups (F = 3.438, p = 0.017). Results from the analysis of the comparison of variances using Levene’s test (F = 7.1, p < 0.001) also infer the signiﬁcant inequality of RT-PCR positivity periods in different age groups in the case of asymptomatic patients and no signiﬁcant difference in RT-PCR positivity periods in different age groups of symptomatic patients. However, the two-way ANOVA test revealed that age groups are not associated with RT-PCR positivity periods (F = 1.12, p = 0.341), but symptomatic and asymptomatic patients are associated with RT-PCR positivity days (F = 66.94, p = 0.00). The interaction effect between the age group, and symptomatic and asymptomatic patients is not statistically significant (F = 1.66, p = 0.174), indicating that the relationship between symptomatic and asymptomatic patients, and RT-PCR positivity periods is not associated with age groups (Figure 3a).

The box plot analysis of RT-PCR positivity among different genders in symptomatic and asymptomatic patients is presented in Figure 2b. One-way ANOVA test showed that in symptomatic patients, there was no significant variation among the different genders (F = 0.4256, p = 0.5), but in asymptomatic patients, significant variation was observed among the genders (F = 4.471, p = 0.03). The results are also supported by Levene’s test. The significant p value of two-tailed analysis of variance (ANOVA) infers that symptomatic and asymptomatic patients are associated with RT-PCR positivity periods (F = 161.67, p = 0.00), but gender is not associated with RT-PCR positivity periods (F = 1.1, p = 0.296). The significant interaction between gender, and symptomatic and asymptomatic patients (F = 3.88, p = 0.049) indicates that the relationship between symptomatic and asymptomatic patients, and RT-PCR positivity periods is associated with gender (Figure 3b).

In symptomatic patients, the mean duration of RT-PCR positivity in hypertensives and non-hypertensives was 15.0 ± 4.6 and 15.5 ± 4.7 days, respectively. The mean duration in diabetics and non-diabetics was 15.8 ± 4.7 and 15.0 ± 4.5 days, respectively. The mean duration in patients with and without malignancy was 17.0 ± 4.8 and 15.2 ± 4.6 days, respectively. In asymptomatic patients, the mean duration of RT-PCR positivity (from the first positive RT-PCR to the last positive RT-PCR) in hypertensive and non-hypertensives was 9.2 ± 3.8 and 8.8 ± 4.3 days, respectively. In diabetics and non-diabetics, the duration was 9.5 ± 4.8 and 8.6 ± 4.1 days, respectively. The mean duration in patients with and without malignancy was 5 ± 1 and 8.9 ± 4.2 days, respectively.

One-way ANOVA test showed that in both symptomatic and asymptomatic patients, there was no significant variation among the different comorbidities at a p value of >0.05. Levene’s post hoc test also signifies no significant variation among the different comorbidities in both symptomatic and asymptomatic patients. Three-way ANOVA analysis showed that the RT-PCR positivity periods are not associated with the presence or absence of comorbidities and types of comorbidities; however, the same is significantly affected by symptomatic and asymptomatic patients (p<0.05). The box plot analysis of RT-PCR positivity associated with different comorbidities in symptomatic and asymptomatic patients is depicted in Figure 2c. The interaction effect between the presence or absence of comorbidities, types of comorbidities, and symptomatic and asymptomatic patients is not statistically significant (p > 0.05), demonstrating that the relationship between symptomatic and asymptomatic patients, the presence or absence of comorbidities, and RT-PCR positivity periods is not associated with the types of comorbidities (Figure 3c). Among the symptomatic patients, 16 (1.07%) had positive RT-PCR beyond three weeks after the onset of symptoms. Of them, two were diabetes mellitus cases, three were hypertensive cases, and 11 were above 60 years of age.

**Figure 4:**
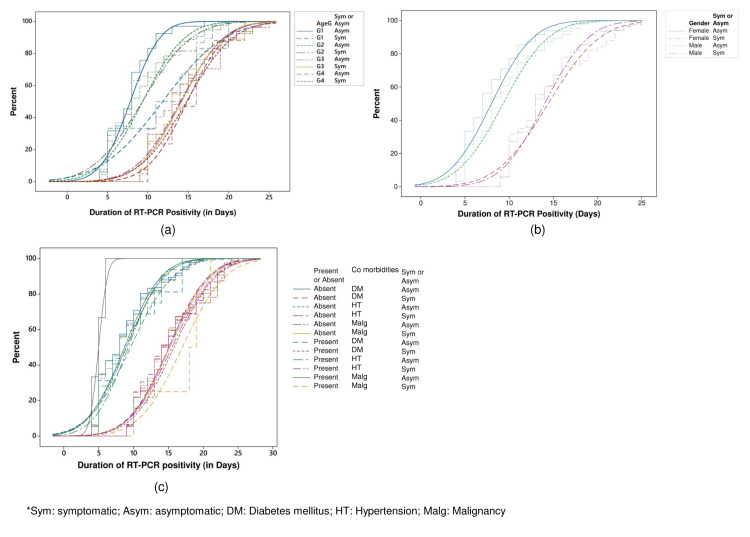
Empirical CDF of (a) age groups, (b) gender, and (c) comorbidities among symptomatic and asymptomatic patients CDF: cumulative distribution function, RT-PCR: real-time polymerase chain reaction, Sym: symptomatic, Asym: asymptomatic, DM: diabetes mellitus, HT: hypertension, Malg: malignancy

The empirical CDF plot analysis highlighted the empirical distribution of age groups, gender, and comorbidities among the patients (Figure 4a-4c).

**Figure 5:**
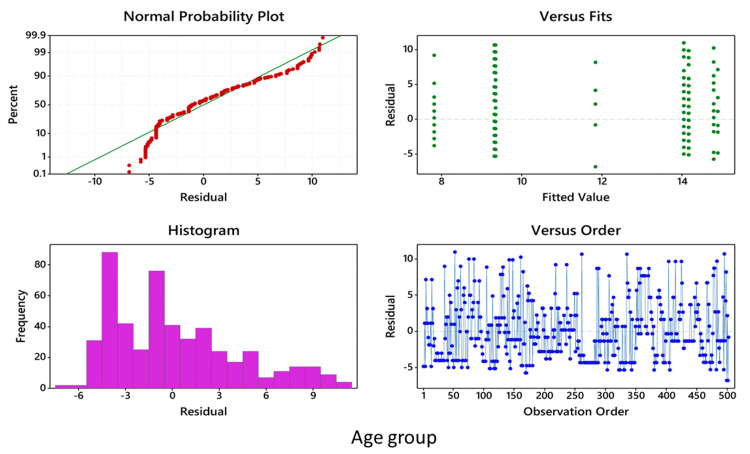
Two-way ANOVA showing the comparison of the symptomatic and asymptomatic groups in relation to age group ANOVA: analysis of variance

**Figure 6:**
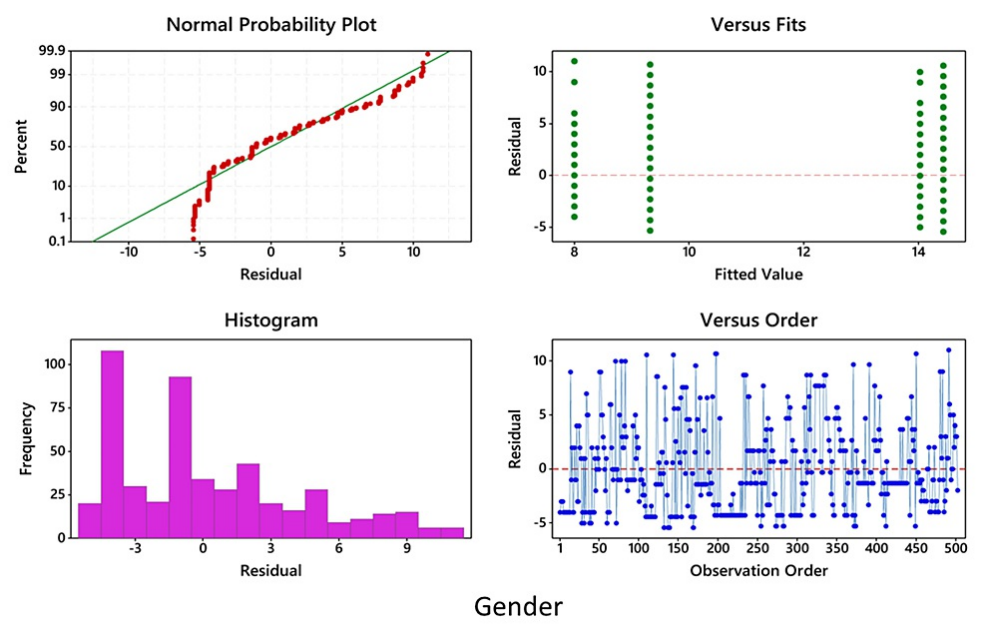
Two-way ANOVA showing the comparison of the symptomatic and asymptomatic groups in relation to gender ANOVA: analysis of variance

**Figure 7:**
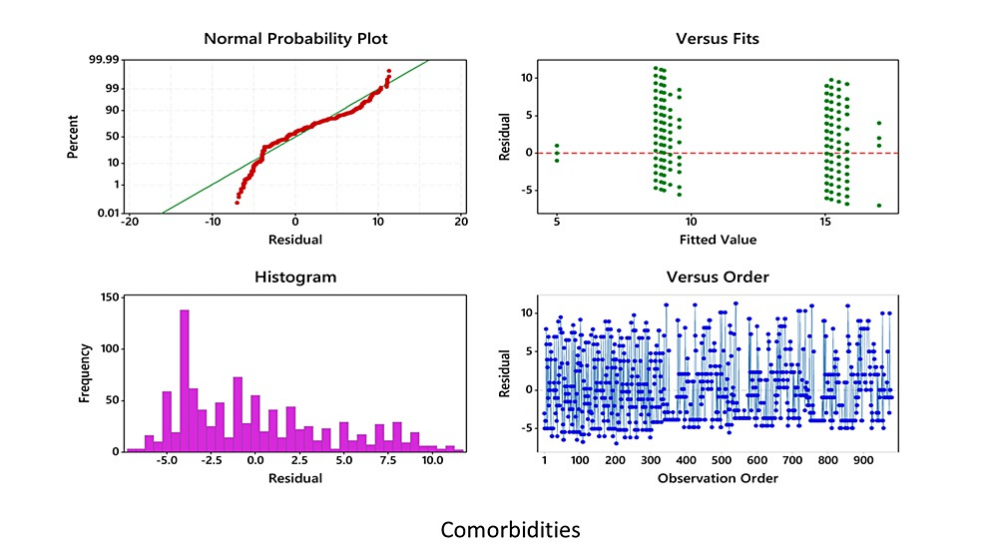
Multi-way ANOVA showing the comparison of the symptomatic and asymptomatic groups in relation to comorbidities ANOVA: analysis of variance

In all cases, before performing ANOVA, residuals versus fits plot, residuals versus order plot, and the normality plot of the residuals were performed, which showed that the data are normally distributed and fall randomly around the center line (Figures 5-7).

The management of the patients was done as per the ICMR guidelines and institutional protocol [9].

## Discussion

This study was conducted in a tertiary care hospital in Assam in Northeast India and included 500 COVID-19 symptomatic and asymptomatic patients with 2,088 SARS-CoV-2 RT-PCR tests for the detection of viral nucleic acid in nasopharyngeal and oropharyngeal samples. The results of this study will provide some significant information regarding the dynamic profile of SARS-CoV-2 in patients of this region and will help understand the impact of demographic and clinical profiles on it. It is important to understand the pathogenesis and transmission dynamics of SARS-CoV-2 for better management of cases and to check its transmission and prevent the further spread of the pandemic. Early detection and isolation of cases are very crucial to halt the transmission of disease. Since the SARS-CoV-2 pandemic has been still continuing, the findings of this study will help in guiding the health authorities in preparing the appropriate protocols for quarantine, contact tracing, and isolation.

In the present study, males predominated the symptomatic cases. Similar findings were reported by Xiao et al. (2020) [10], Bhattacharya et al. (2020) [11], and Alshukry et al. (2020) [12]. The biological differences in the immune systems existing between males and females may influence the ability to fight infection including SARS-CoV-2, and that may be the reason for the male predominance in the study. In general, females are less prone to infections than males. This may possibly be mediated by several factors, such as sex hormones, increased expression of coronavirus receptors (angiotensin-converting enzyme-2 (ACE-2)) in males, and lifestyle differences, such as higher levels of smoking and drinking behavior among males as compared to females. Moreover, females show more responsible attitudes toward COVID-19 than males [13].

Although fever was the most common presenting symptom, it was absent in 42.3% of patients, which implies that the absence of fever at the time of initial screening does not exclude COVID-19. A similar finding was also reported by Khatri et al. (2021) [14]. The absence of fever as an initial clinical presentation may delay early diagnosis of COVID-19, or many a time, the diagnosis may be missed. Also, the diagnosis of COVID-19 is often difficult when the patients are asymptomatic. Therefore, the clinical diagnosis of COVID-19 in symptomatic patients should not be based on the presence of fever as an initial symptom. To avoid delay in diagnosis, contact history, especially household exposure or other contact histories, and other clinical manifestations such as cough, expectoration, polypnea, tightness of chest, and diarrhea should be considered as well. However, as fever seems to be an important initial symptom of COVID-19, to prevent the further spread of the disease in the community, a digital infrared thermometer with maximum accuracy could be considered for mass screening of patients with a history of contact with COVID-19-positive individuals or history of intra- and intercountry traveling [15].

Multiple SARS-CoV-2 variants are circulating globally. Several new variants emerged, most notably the B.1.1.7 lineage (aka 20I/501Y.V1 Variant of Concern (VOC) 202012/01) (United Kingdom), B.1.351 lineage (aka 20H/501Y.V2) (South Africa), P.1 lineage (aka 20J/501Y.V3) (Brazil), B.1.617.2 (Delta, India), and B.1.1.529 (Omicron, South Africa) [16]. VOC cases (B.1.1.7, P.1, and B.1.351) were found to be associated with an increased rate of hospitalization and ICU admission compared to non-VOC cases [17]. On the contrary, a group of researchers found that viral load and disease transmission is higher in B.1.1.7 lineages, but no association could be established between severe disease and infection with the alpha variant [18]. Studies conducted in Scotland and Singapore revealed that hospitalization was more in persons infected with the delta variant, as the oxygen requirement was higher among them and they suffered for a longer duration [19,20].

The study revealed that symptomatic patients had a longer period of RT-PCR positivity from the onset of symptoms. Similar findings were also reported by Bhattacharya et al. (2020) from All India Institute of Medical Sciences, New Delhi [11]. In the present study, the proportions of positive RT-PCR tests were similar to other studies [11]. Xiao et al. reported RT-PCR positivity of 100%, 89.3%, 66.1%, 32.1%, 5.1%, and 0% in the first, second, third, fourth, fifth, and sixth weeks of illness, respectively [10]. The findings of the study suggest that the duration of SARS-CoV-2 viral replication is relatively longer for more than two weeks in >40.6% of infected patients. Hence, it indicates that to control the transmission of the disease, exclusive monitoring of infected patients is required until at least two consecutive negative RT-PCR results are obtained.

The median duration of the virus in respiratory samples was 7-23 days as reported in a meta-analysis of various studies [21]. The present study revealed a shorter median duration of RT-PCR positivity (13 days), which is consistent with other studies [22-24]. Few patients in the study showed detection of SARS-CoV-2 RNA for a prolonged period, i.e., longest duration being 25 days following symptom onset. Xiao et al. also noted similar findings of prolonged viral nucleic acid detection in two published studies [10,25]. It is difficult to establish the exact period of infectivity of COVID-19; however, transmission from asymptomatic patients has been documented earlier. Studies have documented that transmission from asymptomatic individuals was estimated to account for more than half of all transmissions. Identification of asymptomatic carriers is very important for taking public health measures considering the high basic reproductive number (R0->2.5) and high secondary attack rate of SARS-CoV-2 among households and close contacts [26]. In addition to the detection and isolation of symptomatic COVID-19 patients, for the effective control of disease spread, it is important to reduce the risk of transmission from asymptomatic patients.

The impact of the demographic and clinical factors on the duration of RT-PCR positivity from the onset of symptoms or the first PCR was studied and analyzed. There was no significant difference in the duration of RT-PCR positivity within different age groups, gender, and comorbidities (diabetes, hypertension, and malignancies). The information regarding the exact contact history and the time of exposure with a confirmed case is difficult to obtain, limiting the comparison of the duration of RT-PCR positivity between asymptomatic and symptomatic patients. In the present study, no significant difference was noticed when the duration of RT-PCR positivity was compared between the symptomatic and asymptomatic groups. Older patients aged 60 years or more showed a significantly prolonged duration of RT-PCR positivity. Xu et al. studied various factors associated with prolonged viral shedding in a retrospective study, which revealed that older age, male gender, treatment with corticosteroids, delay in hospital admission, and requirement of mechanical ventilation are the associated risk factors [27]. The increased immune dysfunction in older patients may possibly explain the reason for the prolonged persistence of SARS-CoV-2 in their respiratory samples [28]. Hence, in the case of the elderly population, it is desirable to consider prolonged observation and repeat RT-PCR testing before their discharge from the hospital or quarantine facility.

The present study has some limitations. The detection of viral nucleic acid in respiratory samples does not indicate the presence of the viable virus, and therefore, RT-PCR positivity does not necessarily relate to infectivity. La Scola et al. analyzed the correlation between the cycle threshold value (Ct value) of RT-PCR and viral isolation in cell culture, where they found that patients with Ct values ≥ 34 do not release infectious viral particles [29]. Also, in the present study, a test with a Ct value ≤ 34 was considered RT-PCR-positive. However, further studies focusing on the viability of the SARS-CoV-2 virus in respiratory samples are needed to come to a firm conclusion. Also, due to the lack of availability for cell culture, the exact period of infectivity of the patients could not be determined.

## Conclusions

To the best of our knowledge, this is the first study that describes the dynamics of the RT-PCR profile of COVID-19 patients in Assam, a state in Northeast India. According to the findings of the study, RT-PCR tests are to be repeated from respiratory specimens before safe discharge and discontinuation of quarantine. Older age was associated with prolonged RT-PCR positivity, and hence, elderly patients and patients with comorbidities should be considered, especially with regard to management and their containment. To come to a conclusion regarding viral shedding and infectivity, further studies are required to compare RT-PCR results with viral isolation.
